# An adaptable microreactor to investigate the influence of interfaces on *Pseudomonas aeruginosa* biofilm growth

**DOI:** 10.1007/s00253-021-11746-5

**Published:** 2022-01-11

**Authors:** Zhang Ye, Dina M. Silva, Daniela Traini, Paul Young, Shaokoon Cheng, Hui Xin Ong

**Affiliations:** 1 School of Mechanical Engineering, Faculty of Engineering, Macquarie University, Sydney, NSW 2113 Australia; 2 Woolcock Institute of Medical Research, Sydney, Australia; 3 Department of Biomedical Sciences, Faculty of Medicine, Health and Human Sciences, Macquarie University, Sydney, NSW 2113 Australia; 4 Department of Marketing, Macquarie Business School, Macquarie University, Sydney, NSW Australia

**Keywords:** Dual-chamber microreactor, Interfaces, Biofilm, Static culture, Antibiotic susceptibility, MBEC

## Abstract

**Abstract:**

Biofilms are ubiquitous and notoriously difficult to eradicate and control, complicating human infections and industrial and agricultural biofouling. However, most of the study had used the biofilm model that attached to solid surface and developed in liquid submerged environments which generally have neglected the impact of interfaces. In our study, a reusable dual-chamber microreactor with interchangeable porous membranes was developed to establish multiple growth interfaces for biofilm culture and test. Protocol for culturing *Pseudomonas aeruginosa* (PAO1) on the air–liquid interface (ALI) and liquid–liquid interface (LLI) under static environmental conditions for 48 h was optimized using this novel device. This study shows that LLI model biofilms are more susceptible to physical disruption compared to ALI model biofilm. SEM images revealed a unique “dome-shaped” microcolonies morphological feature, which is more distinct on ALI biofilms than LLI. Furthermore, the study showed that ALI and LLI biofilms produced a similar amount of extracellular polymeric substances (EPS). As differences in biofilm structure and properties may lead to different outcomes when using the same eradication approaches, the antimicrobial effect of an antibiotic, ciprofloxacin (CIP), was chosen to test the susceptibility of a 48-h-old *P. aeruginosa* biofilms grown on ALI and LLI. Our results show that the minimum biofilm eradication concentration (MBEC) of 6-h CIP exposure for ALI and LLI biofilms is significantly different, which are 400 μg/mL and 200 μg/mL, respectively. These results highlight the importance of growth interface when developing more targeted biofilm management strategies, and our novel device provides a promising tool that enables manipulation of realistic biofilm growth.

**Key points:**

*• A novel dual-chamber microreactor device that enables the establishment of different interfaces for biofilm culture has been developed.*

*• ALI model biofilms and LLI model biofilms show differences in resistance to physical disruption and antibiotic susceptibility. *

## Introduction

Biofilms are the dominant surviving model of bacteria that exist on earth (Donlan 2002). It plays a crucial part in the ecosystem, either beneficial or detrimental depending on the microbial species and their growth. For example, crops can benefit from non-pathogenic biofilm plant growth-promoting rhizobacteria (PGPR) biofilm (Rudrappa et al. 2008) but can also trigger foodborne illnesses in plant diseases caused by pathogenic microbial biofilms (Buttimer et al. 2017). Also, while biofilms can help degrade pollutants in liquid and gaseous effluents in wastewater treatment plants, undesired biofilm formation in drinking water, oil pipelines, and ship hulls can lead to biofouling and biocorrosion, which undermine operation safety and loss of productivity (Muhammad et al. 2020). Furthermore, biofilms are typically associated with clinical chronic, nosocomial, and medical device-related infections (Khatoon et al. 2018; Stoodley et al. 2013; Sunarintyas 2016; Vickery et al. 2011).

The formation of biofilm starts with planktonic bacteria adhering to the surface and encasing the proliferated colonies in self-produced extracellular polymeric substance and become matured biofilm (Donlan 2002). Biofilms protect the microorganism from hostile physical and chemical environments such as altered pH, osmolarity, nutrients scarcity, and mechanical and shear forces and block bacterial biofilm communities’ access from antibiotics and host’s immune cells (Sharma et al. 2019). Their properties are determined by both inherent biological attributes of bacterial strains and external environmental factors. Among all the environmental factors, surfaces under the presence of different interfaces play a critical role that directly affects bacteria’s initial attachment, biofilm maturation, final detaching, and interaction with environmental factors. However, most of the biofilm studies to date have persistently been modeled as solid-attached structures submerged in liquid (solid–liquid interface, SLI), and the impact of varying growth interfaces have been neglected due to the limitation of current biofilm culture models such as microtiter plate (Kolter and Losick 1998; Martí et al. 2011; Wang et al. 2010; Whitehead and Verran 2009), the Calgary Biofilm Device (Ceri et al. 1999), the center for disease control reactor (Gilmore et al. 2010), the rotating disk reactor (Cotter et al. 2010), and the microfluidic devices (Pousti et al. 2018). In fact, biofilms can grow on surfaces that are subjected to the air–liquid interface (ALI) which when colonized provides bacteria with access to both the gaseous (e.g., oxygen) and liquid (e.g., nutrient) phases or on the liquid–liquid interface (LLI) where biofilm intact to the liquid phases from two sides. For example, biofilms formation in the respiratory system and on the roots or leaves of plants can be modeled as ALI biofilm, while biofilms that developed in the urinary tract, on porous medical indwelling devices, and porous membrane used in wastewater treatment plant can be best characterized as LLI biofilm. Furthermore, antimicrobials have been widely used to treat biofilm infection diseases with multiple delivery paths, including parenteral, enteral, transdermal, inhalation, oral, and topical. *Pseudomonas aeruginosa* is a ubiquitous pathogen that could colonize multiple environments and establish a biofilm within 24 h (Webster et al. 2015) and matured within 48 h (Borriello et al. 2004). It can cause a variety of infections, such as chronic lung infection (Faure et al. 2018) and urinary tract infections (UTIs) (Mittal et al. 2009), and its accentuated antibiotic resistance during biofilm growth poses a significant threat to the medical community. The model that better mimic the biofilm attaching interface will help to investigate the drug efficacy delivered though different ways.

For this purpose, we proposed a dual-chamber microreactor with interchangeable interfaces to study biofilm structure and susceptibility to drugs. Our model brings the advantages of a microfluidic device, which include reduced sample volume and reagent consumption and precise environmental control. Moreover, the curing approach and reversible bonding technique adopted for device fabrication and assembling further help to reduce experimental time and cost and bring greater flexibility for sample manipulation. Compared with conventional antimicrobial susceptibly testing methods, our device can be considered more physiobiological relevant in mimicking the microenvironment that biofilm grows and interacts with antibiotics. In this study, we used this novel device to optimize a protocol for culturing *Pseudomonas aeruginosa* (PAO1) on ALI and LLI, respectively, under static environmental conditions for 48 h. Biofilm susceptibility was also evaluated after 6 h exposure to ciprofloxacin from the substrate side to mimic systemic circulation exposure, which permeates through a physical barrier to reach the biofilm site.

## Materials and methods

### Materials

Polydimethylsiloxane monomer and curing agent (Sylgard 184, Dow Corning) were purchased from Revolution Industrial (Australia). Formlabs® Clear resin V4 was obtained from Core Electronics (Australia). Isopropyl alcohol (IPA) (≥ 99%) and ethanol (80% v/v) were obtained from Chem-Supply Pty Ltd (Australia). Ambersil® polymer remover was purchase from RS Components Pty Ltd (Australia). Hydrophilic porous polyester (PETE) membrane (pore size 0.2 μm, pore density 3 × 108 (pores/cm^2^), open area 9.4%, thickness 10 μm, bubble point 20psi, water flow rate 10 mL/min/cm^2^, airflow rate 3L/min/cm^2^) was purchased from Sterlitech (USA). Translucent silicone rubber (TRANSIL®) was obtained from Barnes Products Pty Ltd (Australia). Tygon tubing (1.59 mm OD × 0.51 mm ID), 23G stainless steel couplers (0.63 mm OD × 0.33 mm ID), and razor-sharp stainless steel biopsy punches (0.5 mm and 1.25 mm OD) were purchased from Darwin Microfluidics (France). Stainless steel screws and nuts were obtained from Small Parts & Bearings (Australia). Blunt needles (22G) and sterile disposable syringes were obtained from Livingstone (Australia). *Pseudomonas aeruginosa* (PAO1 ATCC 15,692) was purchased from the American Type Culture Collection (ATCC, Rockville, USA). Cation-adjusted Mueller–Hinton Broth (CAMHB) was purchased from BD Biosciences (Australia). Phosphate-buffered saline (PBS), LB Broth with agar (Lennox), Alcian blue (1% w/v in acetic acid, 3% v/v pH 2.5), AeraSeal® Film, and formaldehyde solution (≥ 36.0% in water) were purchased from Sigma-Aldrich (Australia). FilmTracer™ LIVE/DEAD® Biofilm viability kit was obtained from Thermo Fisher Scientific (Australia). Ciprofloxacin hydrochloride was supplied by MP (Biomedical Australasia Pty Limited, Australia). Milli-Q water was obtained from Biopak® Polisher system (Merck KGaA, Germany).

### Device design and fabrication

**Fig. 1:**
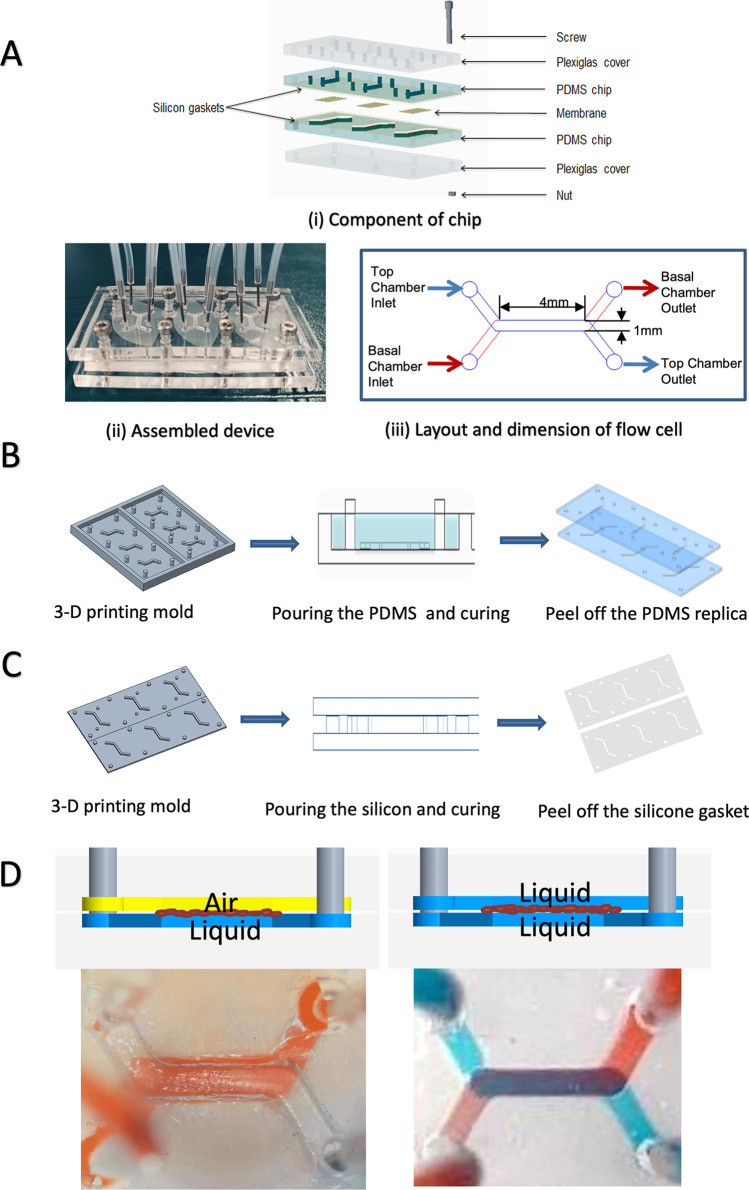
Dual-chamber microreactor fabrication and assembly. **A** Components of chip and assembled device. **B** PDMS chips fabrication process. **C** Silicon gaskets fabrication process. **D** Interface establishment—ALI was established when the bottom chamber was filled with liquid and the top chamber was filled with air, while the LLI was created when both chambers were filled with liquid

The microreactor (Fig. 1) consists of a dual-chamber design (basal and apical) physically separated by a porous hydrophilic PETE membrane and used as the substrate for biofilm growth. Each device comprised three identical dual-chamber flow cells allowing for the testing of multiple experimental setups in parallel. The dimensions of the chamber were 1 mm (width) × 4 mm (length) × 0.5 mm (height). The reactor chambers (Fig. 1B) were fabricated using PDMS by 3D printing microfluidic fabrication technique (Amin et al. 2016). The molds for PDMS casting were printed using a clear resin on a Form2 3D printer (Formlabs), washed for 15 min with IPA (Formlabs, Form wash), and cured under UV light for 90 min at 65 °C (Formlabs, Form Cure). Before pouring the PDMS, the mold was spray-coated with polymer remover to protect the mold surface and to assist with the PDMS removal after the curing process. PDMS monomer and curing agent were mixed (10:1 w/w), cast into the mold, degassed, and cured at 65 °C for 12 h. After cooling, the PDMS chamber was peeled off from the mold, and the inlet and outlet holes were punched using a 0.5-mm biopsy puncher. Two pieces of thin silicon gaskets (0.3 mm) were placed between the membrane and the PDMS layers to prevent leakage and protect the membrane from the mechanical bounding force. The silicone gaskets (Fig. 1C) were fabricated by pouring silicone on the plastic sheet and pressing the 3D-printed template into the uncured silicone mix and against the plastic sheet. The silicone gaskets were cured at room temperate for 25 min and then peeled from the plastic sheet.

### Device assembly

Reversible mechanical bonding of the multiple layers (Fig. 1A) was obtained using two pieces of acrylic Plexiglas covers (76 mm × 25 mm × 3 mm) with holes aligned with the position of the inlet, outlet, and bonding holes in the PDMS layers. The mechanical bonding was achieved by twelve screws and nuts positioned around the perimeter of the reactor chamber to ensure the alignment of the layers and the interface area between the two chambers. The flow into the channels was supplied through microbore tubing connected to stainless steel couplers (23G), inserted into the inlet and outlet ports. Before the bacteria inoculation, 1 mL sterilized water was injected into two chambers to ensure no leaking occurs.

### Device sterilization

Before inoculation, the PDMS chips, acrylic Plexiglas covers, and silicon gaskets were sonicated with Milli-Q water for 480 s, rinsed with an ethanol solution (80% v/v), and exposed to UV light for sterilization for 30 min. The PETE membranes and tubing were sterilized using the autoclave (121 °C/20 min). All the components were assembled aseptically in the biosafety cabinet.

### Device inoculation and biofilm formation

*P. aeruginosa* from frozen stocks was grown on agar plates for 16–18 h at 37 °C. To prepare liquid pre-culture, one colony was transferred into 1 mL of CAMHB media (3 g/L beef extract, 17.5 g/L casein hydrolysate, 1.5 g/L starch, pH 7.0), incubated for 16–18 h at 37 °C, and shaken at 200 revolutions per minute (RPM). The overnight pre-culture was diluted 1:30 v/v in fresh media, incubated for 2 h at 37 °C, and shaken at 200 RPM. The inoculum size was optimized by testing two different standardized densities, which are OD_600_ = 0.04 and 0.4, respectively, to obtain a fully covered membrane within 48 h of growth. Before inoculation, the inoculum size controls were also obtained by viable colony forming units (CFU) counts.

For the device inoculation, 100μL of CAMHB media was injected into the basal chamber through the basal chamber inlet before 100μL of bacterial inoculum was injected into the top chamber through the top channel inlet. After inoculation, the excess media and inoculum were discarded by the tubing removal, and the chip was sealed with a sterile and breathable film (AeraSeal™) and incubated at 37 °C for 2 h under static conditions to allow bacteria attachment. Two different interface models were tested—ALI and LLI. For the ALI establishment, the culture media was removed from the apical chamber after 2 h of bacterial adhesion. For LLI conditions, the apical media was removed after 2 h of attachment and replaced by fresh culture media in the apical chamber. Biofilms were grown at 37 °C for 48 h, and the media on the basal chamber was refreshed after 24 h of culture.

### Device repeatability test

The repeatability of this microreactor was evaluated by quantifying biofilm growth under ALI and LLI models, using CFU counts. Three biological replicates were performed for biofilm formation on ALI and LLI. Two devices containing six flow cells were inoculated for each test model, and another device infused with media was used as controls. Controls for inoculum size were performed after each inoculation by viable colony counts to ensure that differences in the inoculum size were minimal. After 48 h, each membrane was aseptically removed from the device, gently dip-washed in 1 mL of sterile PBS, and subsequently transferred to a tube containing 1 mL of sterile PBS. To disrupt the biofilm, the membranes were sonicated for 280 s at 47 kHz and 1.8 W/cm^2^, previously shown to cause no deleterious effect on the colony-forming ability of *P. aeruginosa* (Suci et al. 1994). Following the disruption, ten-fold serial dilutions of the detached bacterial suspensions were performed in sterile PBS, plated on LB agar plates, and incubated at 37 °C for 16–18 h. Viable colony counts were calculated using Eq. 1:

1 $$CFU/mL=N\times 10/{10}^{-D}$$

where *N* represents the colony number and *D* represents the number of 1:10 dilutions. Six randomly selected membranes were analyzed by scanning electron microscopy (SEM) to confirm that bacteria had been removed from the surface.

### Scanning electron microscopy

The membrane coverage of bacteria attachment and the morphology of the 48-h-old biofilms grown on the devices was visualized using scanning electron microscopy (SEM, JEOL, JMC-6000 NeoScope™ Benchtop SEM, USA) at 15 kV. The devices were disassembled, and the membranes were gently dip-washed in sterilized water before fixation in a formaldehyde solution in PBS (4% v/v) for 90 min. After fixation, membranes were gently rinsed with water and air-dried at room temperature. Before imaging, the membranes were sputter-coated with gold for 2 min using a Smart Coater (JEOL, USA). The images were obtained from two replicates which six images were picked on each replicate. The percentage of bacteria coverage on membrane during the initial attachment period was measured using ImageJ (The National Institutes of Health and the Laboratory for Optical and Computational Instrumentation, University of Wisconsin).

### Confocal laser scanning microscopy (CLSM)

The biofilm formed on different interfaces was investigated using FilmTracer™ LIVE/DEAD® Biofilm viability kit according to the manufacturer’s specifications. The samples were gently washed with sterilized water followed by staining with LIVE/DEAD solution for 25 min at 37 °C in the dark. Excess dye was removed using sterilized water. The biofilms were then fixed for 40 min by infusing PFA solution (4% v/v in PBS). The sample was rinsed with sterile water and mounted on a glass slide. Fluorescence was observed using a confocal laser scanning microscope (Olympus FluoView, inverted FV 3000RS IX83, 100 × magnification oil immersion objective). The image was acquired using 488-nm and 660-nm incident light. The z-stack images were taken with a step of 1 μm, and the resolution was 1,024 × 1,024 pixels. All experiments were performed in duplicate. 3D reconstruction view and 3D z-stack projection images were obtained, and fluorescence intensity was measured using ImageJ. The ratio of live cells to dead cells was compared using the fluorescent intensity ratio of green to red in 3D-projected z-stack CLSM images to quantify the difference between biofilm formed on different interfaces. For analysis, the images were obtained from two replicates which three images were picked on each replicate. Thus, six images in total for each biofilm model were collected for statistical analysis.

### Extracellular polymeric substances (EPS) staining

Alcian blue staining was used as a colorimetric-based approach to detect the polysaccharides in the extracellular polymeric substances (Wu et al. 2020). The membrane was aseptically removed from the device, gently washed with sterilized water, and then fixed using 4% (v/v) formaldehyde solution in PBS for 40 min. The membrane was rinsed with water again and then immersed in Alcian blue (1% w/v in acetic acid, 3% v/v pH 2.5) for 15 min. Excess dye was removed by multiple rinses with water. Images were obtained using NanoZoomer-SQ Digital slide scanner C13140-01 (Hamamatsu). Eight images were collected at 40 × magnification from each sample, and the mean red, green, and blue (RGB) ratio of Alcian blue and standard deviation were obtained using ImageJ.

### Antibiotic treatment test

Ciprofloxacin (CIP) is a broad-spectrum antibiotic that is effective against PAO1 (Brazas and Hancock 2005) and hence has been used as the model antibiotic in this study. To assess the effects of the different interface on *P. aeruginosa* susceptibility to CIP, the 48-h-old biofilms were exposed to a range of concentrations (50–1600 μg/mL) for 6 h. For the antibiotic treatments, a stock solution of CIP was prepared in water, and working solutions were freshly prepared by dilution of the stock in culture media. The antibiotic solution was injected into the basal chamber, and the biofilm interacts with antibiotics from the substrate side for 6 h at 37 °C. The susceptibility was assessed by CFU counts according to the procedure described in the device repeatability test section.

### Statistical analysis

The repeatability of culturing biofilms at different interfaces was examined by calculating the standard deviations across all experiments. All data are expressed as Log (CFU/mL). Unpaired *t*-tests were performed to determine the significant differences between the biofilm formed at ALI and LLI. The antibiotic susceptibility of biofilm formed on ALI and LLI was investigated using one-way ANOVA. Graphs show that means and error bars indicate the standard error of the mean. All the statistical analysis was performed using GraphPad Prism 7.0.

## Results

### Optimization of inoculum concentration and biofilm attachment time

**Fig. 2:**
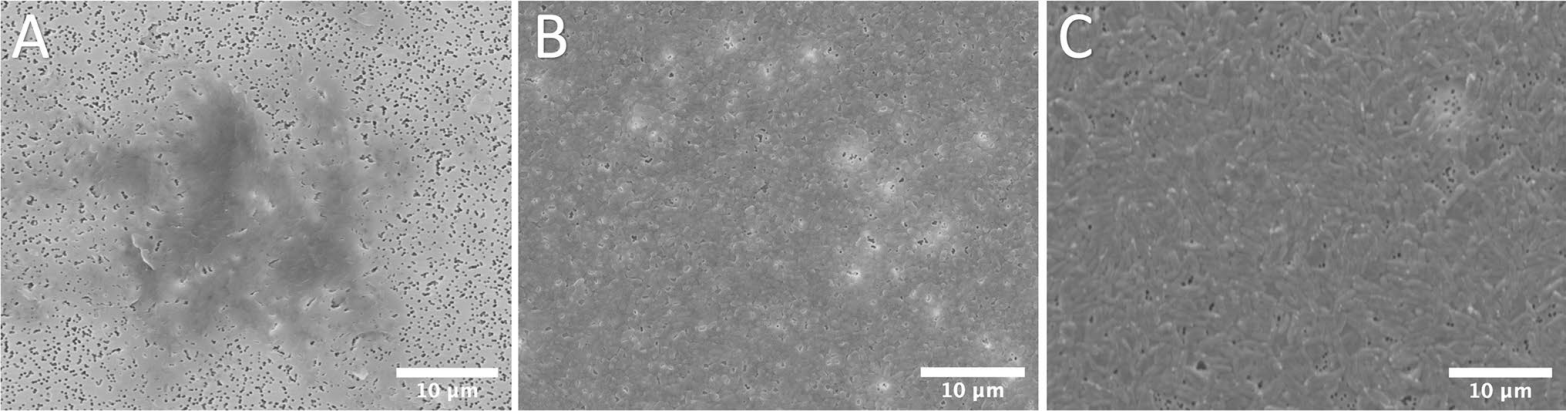
SEM imaging of bacteria attachment using different concentration of inoculum. **A** inoculum size, OD_600_ = 0.04; attachment time, 2 h. **B**) Inoculum size, OD_600_ = 0.04; attachment time, 24 h. **C** Inoculum size, OD_600_ = 0.4; attachment time, 2 h

Inoculum size optimization was performed to establish the formation of a mature biofilm throughout the membrane in 48 h. The investigated inoculum density was standardized to OD_600_ = 0.04 and OD_600_ = 0.4 with inoculation times of 2 h and 24 h. Figure 2 presents the microphotographs of the biofilms grown from different inoculum densities and with different attachment periods. The low-density inoculum (OD_600_ = 0.04) showed that only 8% of the membrane area had bacteria attachment within 2 h and 98% coverage was achieved with 24 h attachment. Biofilm growth from the high-density inoculum (OD_600_ = 0.4) achieved 96% membrane coverage within 2 h of attachment. Thus, inoculum of OD_600_ = 0.4 and 2-h attachment time was chosen as the optimal parameter to carry out the experiments throughout this study.

### Device performance repeatability for biofilm culture

The device performance was further confirmed by testing its repeatability on the formation of 48-h-old biofilms over a determined number of experiments. Three rounds of experiments were performed (biological triplicates), and each round contains six technical replicates. A total of eighteen replicates for each biofilm model (ALI and LLI) were obtained. The inoculum solutions were standardized at OD_600_ = 0.42 ± 0.03 (mean ± SD), which corresponds to Log (CFU/mL) = 8.58 ± 0.14 (mean ± SD). After 48 h of growth, the number of attached cells (biofilm) and the number of detached cells washed off from the membrane after rinsing were obtained by viable colony counts. Results expressed as Log (CFU/mL) and statistical analysis are presented in Table 1 for the ALI and LLI systems. The standard deviation (SD) for detached, attached, and total biofilm cultured on ALI was 0.99, 0.87, and 0.97 log CFU/mL, respectively, and was 0.98, 0.95, and 0.91 for LLI, respectively. To our knowledge, no comparable data is available since other microdevices are unable of collecting CFU numbers due to the constrain related to the nature of other devices that cannot be disassembled without damaging the cells.

**Table 1:** Summary statistics of the number of sessile cells recovered from membranes after biofilm formation on ALI and LLI model surfaces

	ALI			LLI		
	Detached	Attached	Total	Detached	Attached	Total
Mean	6.06	7.19	7.24	6.23	6.94	7.15
SD	0.99	0.87	0.87	0.98	0.95	0.91
SEM	0.24	0.20	0.20	0.23	0.22	0.22

Detached = cells washed off by rinsing; attached = cells remaining on the membrane after washing and detached from the membrane after sonication; total = the sum of detached and attached. The mean value, standard deviation (SD), and standard error of mean results (SEM) are expressed as Log(CFU/mL) of *n* = 18

### Characterization of biofilms grown under ALI and LLI

**Fig. 3:**
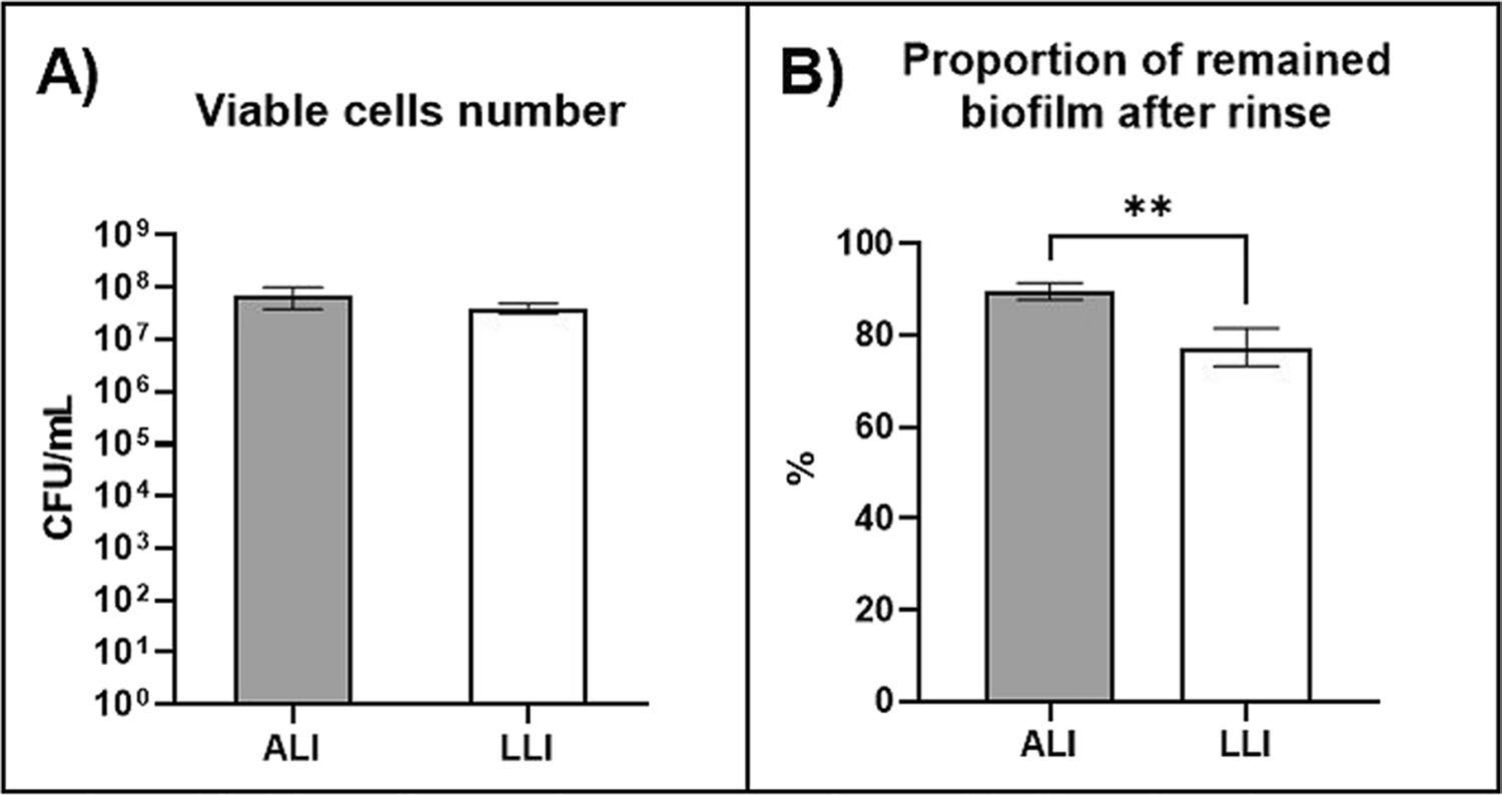
**A** Comparison of bacteria number of intact biofilms formed at ALI and LLI and **B** comparison of the percentage of the remained biofilm after rinse to the intact biofilm (*n* = 18, mean ± SEM) **P < 0.01 (using unpaired *t*-tests)

The differences between the biofilms formed on ALI and LLI in resisting physical disruption of gentle rinse were investigated by analyzing the CFU number of the intact biofilm and the remaining biofilm after rinsing interruption. Figure 3 A and B show the comparison of the CFU number of intact biofilms and the ratio of remained biofilm bacteria to the intact biofilm bacteria, respectively. No significant differences were observed for the bacteria number of intact samples between the ALI biofilm and LLI biofilm. However, the proportion of remained bacteria is significantly higher for biofilms growing on ALI. This indicates that biofilms grown on ALI are more resilient and more difficult to detach from the membrane under gentle washing. Conversely, biofilms grown on LLI are more vulnerable. Hence, the percentage of rinsed-off bacteria is greater. Also, the variability for biofilms grown on LLI is wider, suggesting that these biofilms are more heterogeneous.

**Fig. 4:**
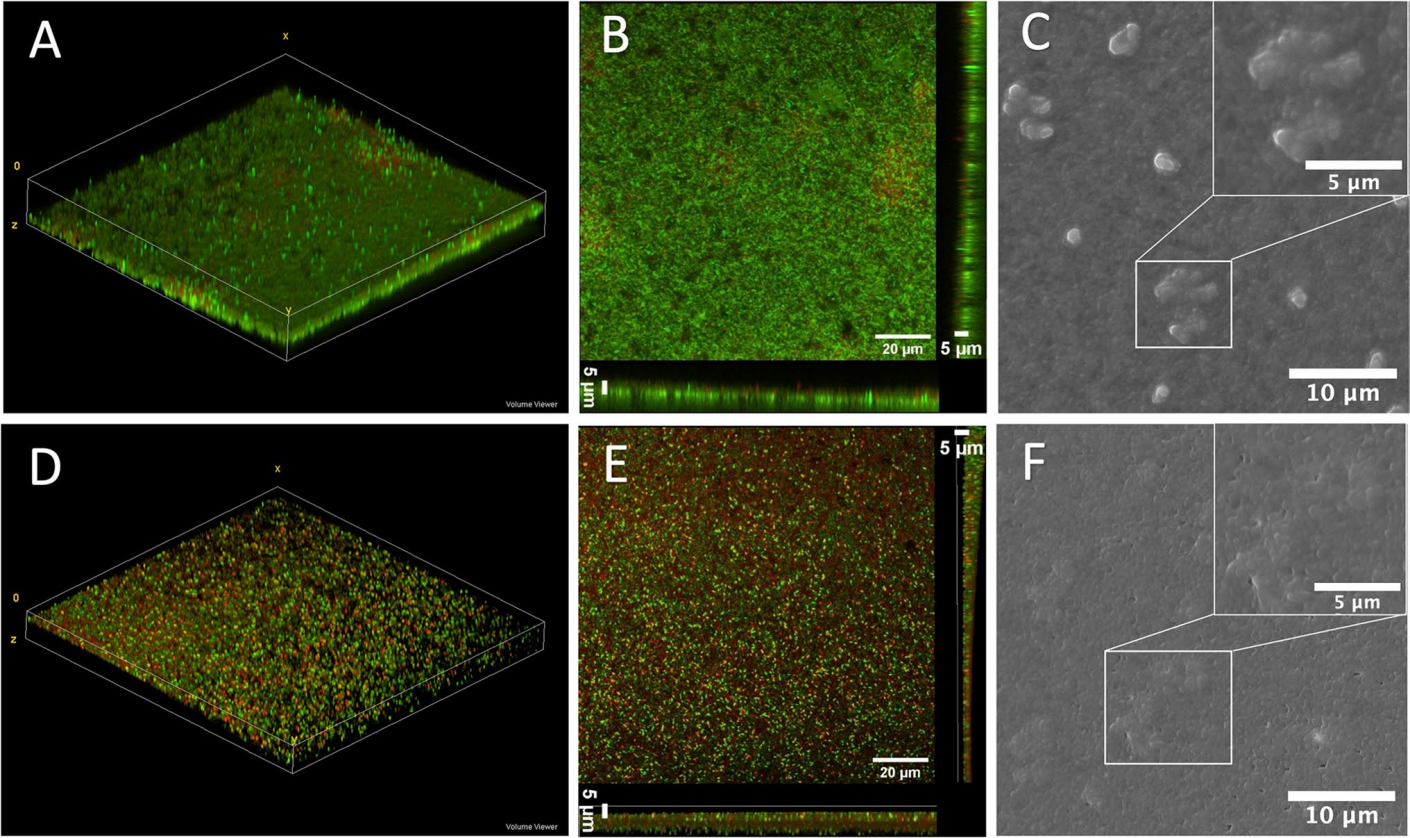
Morphology of biofilms grown under ALI (**A**–**C**) and LLI (**D**–**F**) interfaces: **A** and **D** representative 3D reconstruction of z-stack CLSM images. **B** and **E** 3D projected and orthoclase views of z-stacks obtained from the CLSM images. The live bacteria were stained with SYTO9 (green), and dead bacteria were stained red-fluorescent propidium iodide. **C** and **F** Representative SEM images of PAO1 biofilms showing the “dome-shaped” aggregates; the upper right corner is magnified “dome-shaped” aggregates

The morphology of the 48-h-old ALI and LLI PAO1 biofilms were also visualized using CLSM and SEM (Fig. 4). The 3D reconstruction of z-stack CLSM images (Fig. 4 A and D) show that a layer of biofilm was formed over the membrane and the thickness of the biofilm was approximately 5 μm (Fig. 4 B and E). Dome-shaped bacterial aggregates dispersed across the membrane were observed in SEM images in both ALI and LLI models for PAO1 biofilms (Fig. 4 C and F), and their shapes are more distinct in the ALI compared to the LLI.

In addition, biofilms have also been known to have open water channels that facilitate the diffusion of nutrients and waste products between the external environment and biofilm and within the biofilm matrix [8]. These channels are also evident in SEM images (black holes in the biofilm). Comparing Fig. 4 B and E, the structure of the LLI model biofilm is more dispersed, while the ALI model biofilm is more aggregated. Also, more water channel holes can be seen in the LLI model biofilm SEM images.

**Fig. 5:**
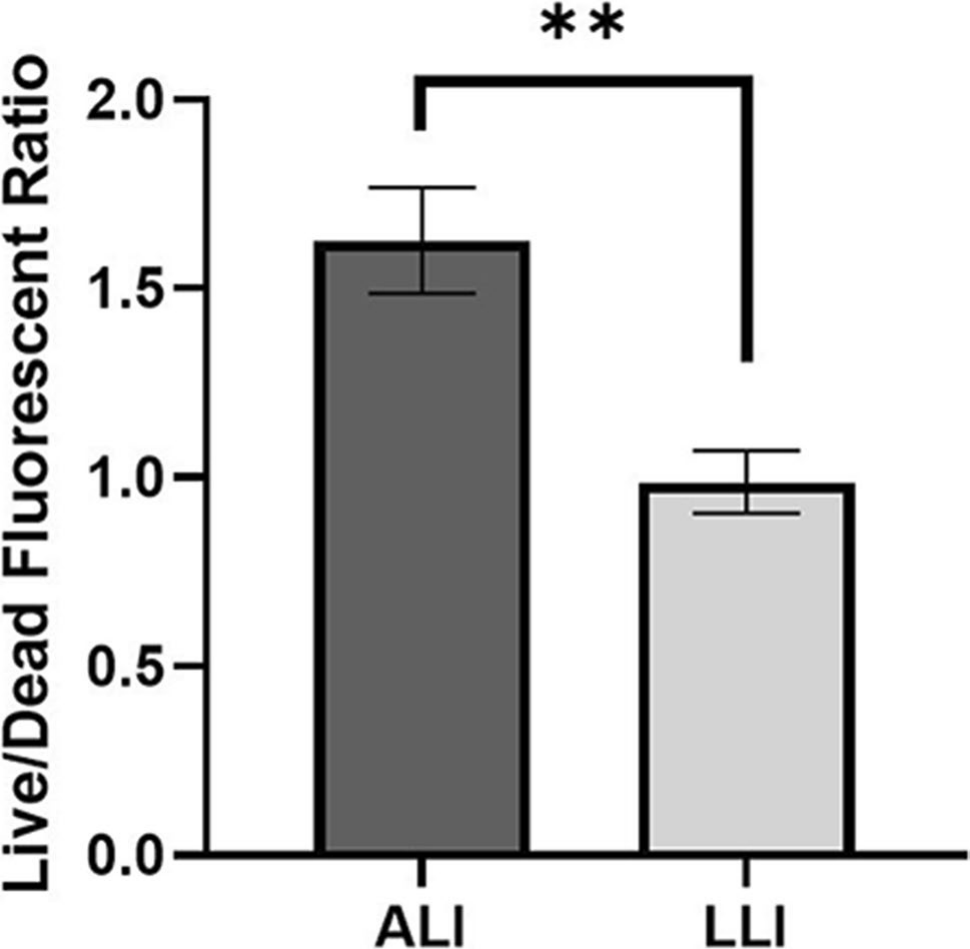
Comparison of the live (green)/dead (red) fluorescent ratio of biofilm formed on ALI and LLI models (*n* = 6, mean ± SEM) **P < 0.01 (using unpaired *t*-tests)

During biofilm formation, both live and dead bacteria are encased within the EPS matrix. The CLSM images (Fig. 4) show a distinct qualitative difference between ALI and LLI biofilms in terms of the amount of live and dead cell. More specifically, biofilm grown on ALI had a higher ratio of living bacteria than the biofilm formed at LLI (Fig. 5). This result corroborates with our previous results in Fig. 3, showing that biofilms formed at LLI were more susceptible to the physical disturbance compared to ALI cultured biofilm, leaving more bacteria on the membrane for ALI model biofilm.

### Extracellular polymeric substances staining

**Fig. 6:**
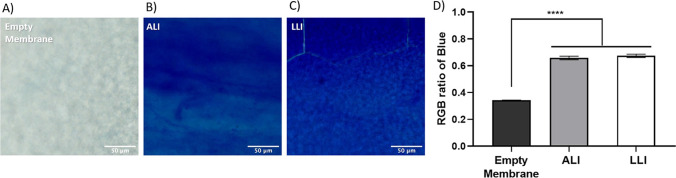
Representative microscopic images of Alcian blue stained samples: **A** empty membrane, **B** ALI biofilm, **C** LLI biofilm, and **D** comparison of the RGB ratio of blue obtained from microscopic images (*n* = 8, mean ± SEM). ****P < 0.0001 (using one-way ANOVA tests)

Alcian blue was used to stain polysaccharides in EPS to provide semi-quantitative data for biofilm formed on both the ALI and LLI models. The RGB ratio obtained from bright-field images of the stained biofilms is depicted in Fig. 6. As the media contains starch, a relatively low blue ratio (≈0.3) was detected in the control group (membrane only). The RGB ratio for biofilm samples is significantly higher than the control sample, indicating that the biofilm has formed on the membrane. However, no significant difference between the ALI and LLI was present, suggesting that the quantity of EPS produced by different biofilm models is similar.

### Antibiotic treatment test

**Fig. 7:**
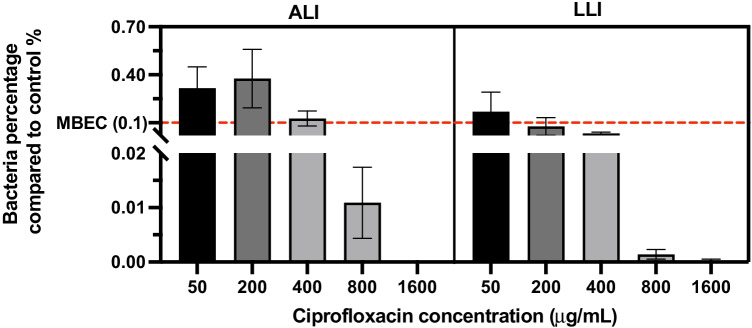
Comparison of the percentage of survived biofilm bacteria number to the growth control group after biofilm samples exposure to CIP (50, 200, 400, 800, and 1600 μg/mL) for 6 h. Antibiotic exposure was performed from the basal chamber (*n* = 6, mean ± SEM). The red dash line indicates the level of MBEC (0.1% bacteria survived after antibiotic exposure)

**Fig. 8:**
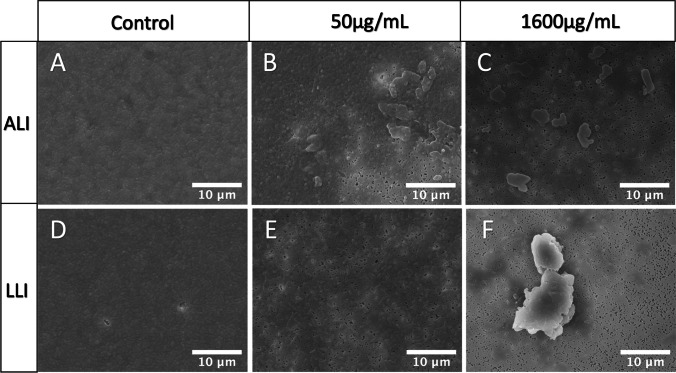
Representative microphotographs of 48-h-old biofilms exposed to CIP for 6 h: **A** untreated biofilm grown in ALI; **B** and **C** biofilms grown in ALI and treated with 50 µg/mL and 1600 µg/mL of CIP, respectively; **D** untreated biofilm was grown in LLI; **E** and **F** biofilms grown in LLI and treated with 50 µg/mL and 1600 µg/mL of CIP, respectively

The results of the antibiotic susceptibility test in the range of 50–1600 μg/mL for the ALI and LLI interfaces are depicted in Fig. 7. Data was presented in the percentage of bacteria number (CFU/mL) recovered from the CIP-treated biofilm sample to the bacteria number of the control group (CFU/mL). In general, CIP induced a concentration-dependent decrease for the biofilm of both interfaces. ALI biofilms appeared more resistant to CIP, showing a larger percentage of bacteria survived than LLI biofilms after being treated with the same CIP concentration. Biofilms were not eradicated at the highest concentration tested (1600 μg/mL). The antibiotic effect on the biofilms was further corroborated by the SEM images (Fig. 8), showing the differences in biofilm density across the membranes when compared to the control (without antibiotic).

The MBEC is defined as the lowest concentration of antimicrobial that eradicates 99.9% of the bacteria in a biofilm state compared with growth controls in the same conditions (0.1% bacteria survived) (Barry 1999). According to this, the MBEC of CIP assessed by our test for 48 h *P. aeruginosa* biofilm grown on ALI and LLI is 400 and 200 μg/mL, respectively.

## Discussion

Biofilms can adapt their architecture to cope with different hydrodynamic conditions and nutrient availability (Teodósio et al. 2011). The interface on which the biofilm grows specifically determines the availability and permeability of nutrients and thus will have an impact on the morphology and properties of the biofilm. Several studies have attempted to investigate the properties of biofilm formed on a particular interface (Rühs et al. 2014; Wu et al. 2012). However, these studies are unable to provide comparative studies on the effect of the interface type using a single platform to control the variables, as most of the instruments are designed to investigate only one specific interface. In this study, an adaptable dual-chamber microreactor has been developed that enables the incorporation of different surfaces that could be subjected to various growth interfaces and to investigate their effects on biofilm development in terms of morphological properties, colony counts, and responses to antibiotics.

The microreactor is fabricated with 3D printing technique (Amin et al. 2016) using affordable equipment and straightforward protocols. PDMS is the ideal material for the fabrication of microdevices having great biocompatibility, chemical stability, gas permeability, transparency, low cost, and ease of molding (Palmer and Caldwell 1995). The mechanical bonding offers more flexibility and convenience for end-point sample manipulation and allows the device to be reused after sterilization which reduce the cost and time of production.

Using this novel dual-chamber microreactor, we have developed the protocol for culturing *Pseudomonas aeruginosa* biofilm on ALI and LLI and adopted several accessible laboratory analysis approaches to investigate the property variance among cultured biofilm samples. The standard deviation values shown in statistical analysis results demonstrate good repeatability of our protocol.

Under defined nutrient supply and cultured in the stagnant environment, the biofilm formed on ALI and LLI developed a similar amount of bacteria and was encased in a similar quantity of EPS. This indicates that nutrient resource is a major factor that determines the proliferation of bacteria and secretion of EPS for forming biofilm structure. Compared to LLI biofilm, ALI biofilm has more distinct “dome-shaped” aggregates and less water channel structure, which we hypothesize that the media transmission occurring in the LLI model is higher than the ALI model, enabling more water channels to be formed to facilitate the transport of nutrients and waste. Furthermore, differences in the structure of the biofilm matrix could contribute to the differences in mechanical properties, which has been demonstrated through the scattered structures (LLI model biofilm), making it more vulnerable to disruption of physical forces. Consequently, the adhesion strength between the biofilm and the underlaid surface and the cohesive force within the biofilm is weaker for the LLI biofilm relative to the ALI biofilm. This further supports the findings in Fig. 3 that LLI biofilms are more susceptible to physical removal.

Different MBEC of CIP outcomes were obtained for ALI and LLI biofilms, which are 400 and 200 μg/mL, respectively, suggesting structural or biological differences among biofilms formed on different interfaces, which could lead to different outcomes while using the same eradication approaches. This highlights the importance of having a model that can closely mimic the environmental conditions where these biofilm communities persist. However, current methods to test antibiotic efficacy are routinely performed with biofilms formed on SLI (Ceri et al. 1999; Khan et al. 2019; Kolter and Losick 1998; Martí et al. 2011; Wang et al. 2010; Whitehead and Verran 2009) with a reported MBEC of 64 μg/mL CIP on a 3-day PAO1 biofilm grown on the microtiter plate method (Wang et al. 2010). While our results showed that significantly higher MBEC of CIP is needed to eradicate biofilms demonstrating that the dual-chamber microreactor could offer an alternative method to study antibiotic susceptibility in a more realistic manner. In this study, the antibiotic solution was injected into the basal chamber and interacts with biofilm from the substrate attaching side by diffusion. This could be treated as a way to mimic the antimicrobial mechanism that the drug being administrated through oral or parenteral routes and taken to the biofilm site through the systemic circulation.

In conclusion, our novel dual-chamber microreactor is able to establish multiple interfaces to better mimic the real biofilm growth condition and provide diverse ways for antibiotic exposure, which could shed light on designing more effective and targeted biofilm control methods. In addition, this dual-chamber microreactor could be further developed into a versatile platform that is able to create more comprehensive dynamic environments for culturing biofilm, which will be the scope of future studies.

## Data Availability

The datasets generated during and/or analyzed during the current study are available from the corresponding author on reasonable request.
